# Supplementary material for: Discovering epistatic feature interactions from neural network models of regulatory DNA sequences
Source: Bioinformatics. 2018 Sep 8;34(17):i629–37. doi: 10.1093/bioinformatics/bty575 (PMC6129272; doi:10.1093/bioinformatics/bty575)
Supplement: Supplementary Data [file bty575_supp.zip › bty575-suppl_data/Supplement.pdf]

SUPPLEMENTARY METHODS

Discovering epistatic feature interactions from neural network models of regulatory DNA sequences

Peyton Greenside, Tyler Shimko, Polly Fordyce, Anshul Kundaje
Stanford University
Contact: pgreens@stanford.edu , akundaje@stanford.edu

A. Benchmarking Feature Interaction Scores on ground-truth motif interactions embedded in simulated regulatory DNA sequences

A.1 Simulating regulatory DNA sequences: We simulated regulatory DNA sequences using the simdna package (https://github.com/kundajelab/simdna). Simulated sequences were 200 bp in length. Nucleotides at each position were sampled independently and randomly from a distribution of [0.27, 0.23, 0.23, 0.27] for A, C, G and T respectively. We simulated 60,000 sequences that were divided into three Sets of 20,000 each (Figure 2A).

We randomly embedded 1 or 2 motif instances of the ELF1 transcription factor (TF) in each sequence in Set 1. The embedded motif sequence was the highest affinity sequence from a known ELF1 Position Weight Matrix (PWM), called "ELF1_known2" in Kheradpour *et al.*[1]. The motif embedding positions were randomly sampled (based on a uniform distribution) across the entire sequence. If a location was sampled that already had a motif embedded, the location was re-sampled until there was sufficient room for the new motif to be embedded. The number of motif instances in each sequence was determined by sampling from a Poisson distribution with mean 2 but allowing only 1 or 2 instances in each sequence. For each sequence in Set 2, 1 or 2 motif instances from the "SIX5_known1" PWM of the SIX5 TF were embedded using the same protocol as for Set 1. For each sequence in Set 3, 1 or 2 motif instances of ELF1 and 1 or 2 motif instances of SIX5 were independently embedded using the same protocol. Hence, all sequences in Set 3 contained both ELF1 and SIX5 motifs. We further independently embedded 0 or 1 instances of the AP1 motif (called "AP1_disc3" in Kheradpour *et al.*[1]) and TAL1 motif (called "TAL1_known1" in Kheradpour *et al.*[1]) in sequences from all three Sets.

A.2 Convolutional neural network (CNN) model trained on simulated regulatory DNA sequences: We set up a binary classification task where all sequences in Set 3 (ELF1 and SIX5) were labeled as positive and all other sequences from Sets 1 and 2 were labeled as negatives. The 60K sequences from Sets 1, 2 and 3 were split into 40K, 10K and 10K subsets to be used as the training, validation and test set respectively.

We used the Keras deep learning framework (https://github.com/keras-team/keras) to train a CNN to classify the sequences. The sequences were represented using a one-hot encoding with 4 channels (A, C, G and T). The CNN architecture is as follows: Layer 1 is a convolutional layer with 40 filters of size 19 and ReLU activation operating on one-hot encoded input sequences. Layer 2 is a max pooling layer of pool length 10. Layer 3 is a fully connected layer of size 200 with dropout (p=0.5) and ReLU activation. Layer 4 is a fully connected layer with a sigmoid activation. The model was trained with the Adam optimizer and binary cross-entropy loss until no improvement was seen for 3 epochs on the validation set. The datasets, code and model are available at https://github.com/kundajelab/dfim

A.3 DeepLIFT nucleotide importance scores for the simulated regulatory DNA sequences: We computed importance scores for each nucleotide in each of the 60K sequences using DeepLIFT[2]. DeepLIFT importance scores quantify the sensitivity of the output logit to finite changes in the input sequence relative to a reference sequence. We used a 4-channel probabilistic reference sequence R of length 200, with $R[(A,T),p] = 0.27$ and $R[(G,C),p] = 0.23$ for each position $p \in \{1 \dots 200\}$. These probabilities match the background nucleotide frequencies used to simulate the regulatory DNA sequences.

B. Uncovering epistatic motif interactions of co-binding transcription factors (TFs) from CNN models of *in vivo* TF binding

B.1 TF ChIP-seq and DNase-seq datasets: We downloaded pre-processed peak calls (binding locations) in hg19 genome coordinates obtained from ENCODE[3,4] ChIP-seq data for three TFs GATA1, GATA2 and TAL1 in the K562 cell-line The URLs for the peak files are
GATA2:
http://hgdownload.soe.ucsc.edu/goldenPath/hg19/encodeDCC/wgEncodeAwgTfbsUniform/wgEncodeAwgTfbsHaibK562Gata2sc267Pcr1xUniPk.narrowPeak.gz
GATA1:
http://hgdownload.soe.ucsc.edu/goldenPath/hg19/encodeDCC/wgEncodeAwgTfbsUniform/wgEncodeAwgTfbsSydhK562Gata1UcdUniPk.narrowPeak.gz
TAL1:
http://hgdownload.soe.ucsc.edu/goldenPath/hg19/encodeDCC/wgEncodeAwgTfbsUniform/wgEncodeAwgTfbsSydhK562Tal1sc12984IggmusUniPk.narrowPeak.gz

FASTQs files for DNase-seq data in K562 were downloaded from the ENCODE[5] portal https://www.encodeproject.org/experiments/ENCSR000EOY/ . The data processing can be exactly reproduced by using our processing pipeline at https://github.com/kundajelab/atac_dnase_pipelines. Briefly, reads from both replicates are mapped to the hg19 human reference genome using Bowtie2. After filtering duplicates and multi-mapping reads, peaks are called for each replicate and for reads pooled from both replicates using MACS2[6] with p-value threshold of 0.01. We also randomly split the reads from the pooled replicates into two equally sized pseudoreplicates. Peaks are called using MACS2[6] on these pseudoreplicates as well. We only retain reproducible peaks from the pooled-replicates that are present in either both individual replicates or present in both pseudo-replicates.

B.2 Multi-task Convolutional neural network (CNN) models of TAL1, GATA1 and GATA2 TF binding in K562: We trained a multi-task CNN to model DNA sequence determinants of *in vivo* binding of the TAL1, GATA1 and GATA2 TFs in the K562 cell line. Each task was set up as a binary classification problem to classify 1kb sequences centered at the TF ChIP-seq peak summits of each TF (positive class) from 1Kb sequences centered at the peak summits of all chromatin accessible DNase-seq peaks (negative class) in K562 that did not overlap the TF's ChIP-seq peaks. For each task (TF), all positive examples overlapped the factor's ChIP-seq peaks and DNase-seq peaks. Negative examples overlapped DNase-seq peaks but not ChIP-seq peaks.

We used the Keras deep learning framework (https://github.com/keras-team/keras) for training the CNN. The sequences were represented using a one-hot encoding with 4 channels (A, C, G and T). The architecture of the CNN model is as follows: Layers 1-5 consist of convolutional layers each with 25 convolutional filters of size 10 and ReLU activations. Layer 6 is a max pooling layer with stride 25. Layer 7 is a final fully connected layer for each task with a sigmoid activation. We held out all examples on chromosomes 8 and 9 for our testing set and used the rest of the data for training and validation. The model was trained with the Adam optimizer and binary cross-entropy loss. Our model achieved mean auROC of 0.953 and mean auPRC of 0.459 on the held-out test set across all three tasks. The datasets, code and model are available at https://github.com/kundajelab/dfim.

B.3 GATA1 and TAL1 motif instances for DFIM analysis: For determining motif sites to compare FIS between putative TAL1 and GATA1 binding sites, we found all exact matches in the input sequences underlying GATA1 and TAL1 ChIP-seq peaks to the pattern 'GATA' for GATA1 and to 'CA**TG' for TAL1 where * can be any base {A,C,G,T}. While we do not expect every such location to be bound by its corresponding TF, these motifs greatly enrich for bound sites of these factors relative to the rest of the sequence. We computed DFIM by mutating the GATA1 motif locations and assessing the FIS of all TAL1 motif locations. We also performed the reverse procedure of mutating TAL1 and found a similar effect on GATA1 motifs. When multiple TAL1 and/or GATA1 motifs appeared in the sequence, we found all combinations of a single TAL1 location and a single GATA1 location and performed the analysis for each pair of locations while holding the rest of the sequence fixed.

C. Discovering interactions between regulatory variants (bindingQTLs) and their target TF motifs from CNN models of *in vivo* chromatin accessibility

C.1 Chromatin accessibility datasets: We obtained FASTQ files for ATAC-seq datasets in 16 primary hematopoietic cell types from Corces *et al.* [7] (available through GEO accession (GSE74912). We also obtained FASTQ files for DNase-seq data in the GM12878 (https://www.encodeproject.org/experiments/ENCSR000EMT/) and K562 (https://www.encodeproject.org/experiments/ENCSR000EOY/) cell lines from the ENCODE[5] portal. The raw fastq files were processed using our ATAC/DNase processing pipeline available at: https://github.com/kundajelab/atac_dnase_pipelines. Briefly, reads from all replicates in each cell type were mapped to the hg19 human reference genome using Bowtie2. After filtering duplicates and multi-mapping reads, we randomly subsampled 50M reads. Peaks are called for each replicate and for reads pooled from both replicates using MACS2[6] with p-value threshold of 0.01. We also randomly split the reads from the pooled replicates into two equally sized pseudoreplicates. Peaks are called using MACS2[6] on these pseudoreplicates as well. Reproducible peaks were called for cell type using the Irreproducible Discovery Rate framework (IDR < 5%).

C.2 Multi-task Convolutional neural network (CNN) models of chromatin accessibility in 18 hematopoietic cell types/cell-lines: We trained an 18-task CNN to model the DNA sequence determinants of chromatin accessibility (measured by either ATAC-seq or DNase-seq) in the 16 primary cells and 2 cell-lines. Each task was modeled as binary classification problem. For each task (cell-type), positive examples consisted of 1Kb DNA sequences overlapping the IDR reproducible DNase-seq/ATAC-seq peaks from that cell type. The negative set for each task consisted of 1Kb DNA sequences overlapping the union of peaks from all 18 cell types, excluding the positive examples for that task.

We used the Keras deep learning framework (https://github.com/keras-team/keras) for training the CNN. The sequences were represented using a one-hot encoding with 4 channels (A, C, G and T). The architecture of the CNN is as follows: Layer 1 is a convolutional layer with 300 filters of length 19 with ReLU activation and Batch Normalization. Layer 2 is a max pooling layer with pooling width 3. Layer 3 is a convolutional layer with 200 filters of length 11 with ReLU activation and Batch Normalization. Layer 4 is a max pooling layer with pooling width 4. Layer 5 is a convolutional layer with 200 filters of length 7 followed by ReLU activation and Batch Normalization. Layer 6 is a max pooling layer with pooling width 4. Layer 7 and 8 are two fully connected layers of size 1000 with batch normalization and dropout (p=0.3) after each layer. Layer 9 for each task is a fully connected layer with sigmoid activation. We initialized our model with weights learned from a multi-task model pre-trained on 900 reference DNase-seq samples from the ENCODE[5] and Roadmap Epigenomics projects[8]. The reference DNase-seq datasets were pre-processed using our ATAC/DNase processing pipeline available at: https://github.com/kundajelab/atac_dnase_pipelines. The pre-trained model is available at https://github.com/kundajelab/dfim. We held out all examples on chromosomes 8 and 9 for our testing set and used the rest of the data for training and validation. We trained the model with the Adam optimizer and binary cross-entropy loss. The model achieved a mean auROC of 0.9 and a mean auPRC of 0.69 across all 18 tasks on the test set. The datasets, code and models are available at https://github.com/kundajelab/dfim

C.3 bindingQTL analysis: We restricted DFIM analysis of significant bQTLs and other control SNVs from Tehranchi *et al.*[9] that overlapped the ATAC-seq/DNase-seq peaks in any of the 18 cell types. We computed DeepLIFT and DFIM Feature Interaction Scores using the GM12878 (lymphoblastoid cell-line) task of our model since the allelic effects of the bQTLs were estimated from ChIP-seq data in pooled lymphoblastoid lines.

Supplementary Figures

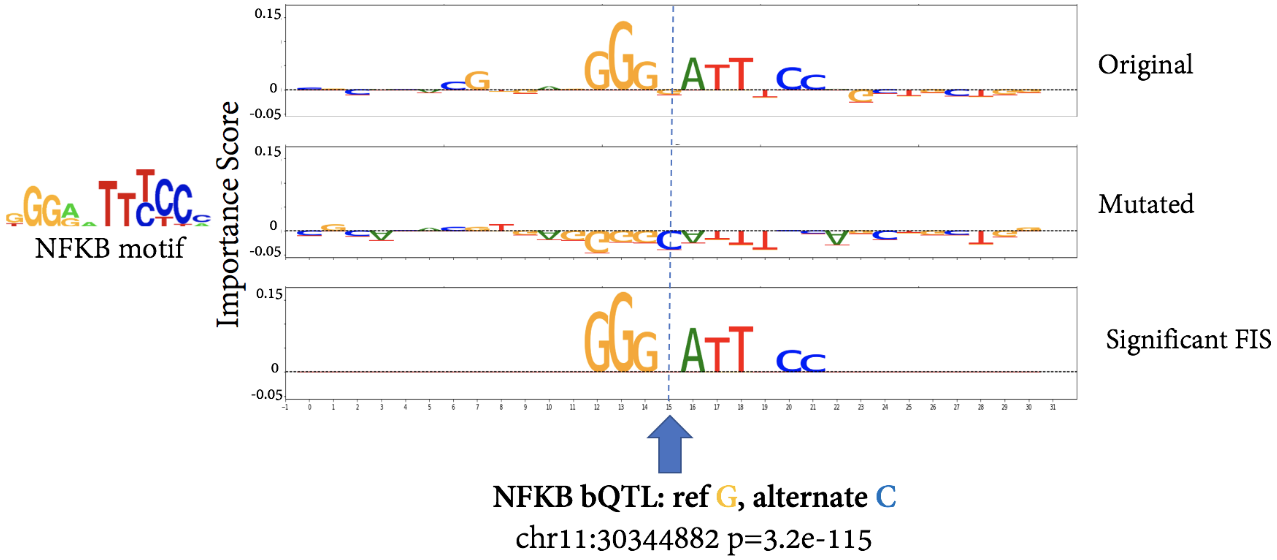

SFig. 1 The NFKB QTL at chr11:30344883 interrupts a known NFKB binding site. The original importance scores are in the top row, the mutated scores after converting the reference allele “G” to a “C.” The delta profile is pictured in the bottom row where all non-significant bases (p>0.05) have been omitted leaving just the responding motif.

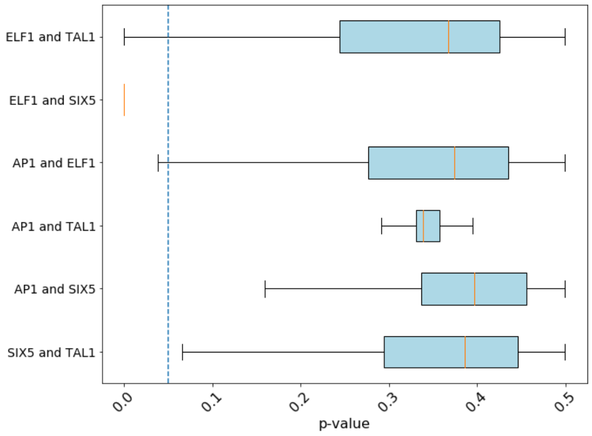

SFig. 2A P-values determined from fitting a NULL distribution to dinucleotide shuffled sequences using DeepLIFT with a fixed GC reference for computing importance scores.

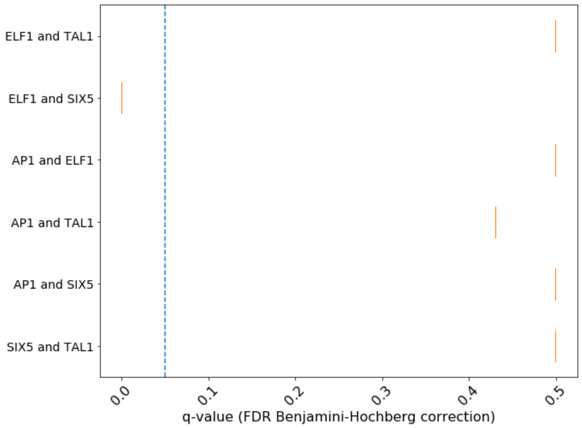

SFig. 2B Q-values – corrected with the Benjamini-Hochberg FDR procedure – of **SFig. 2A.**

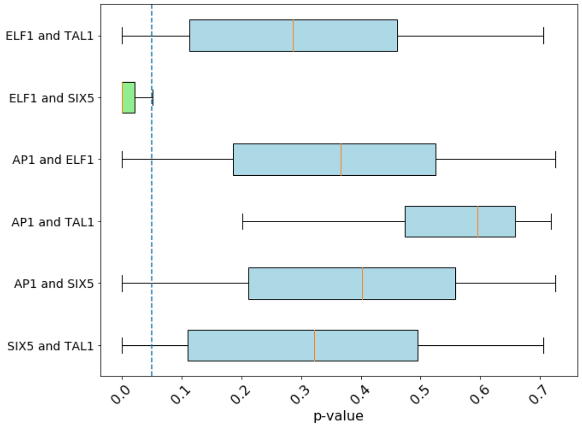

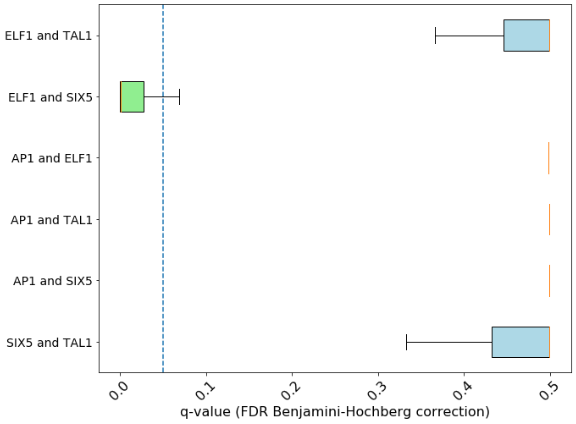

SFig. 2C P-values determined from fitting a NULL distribution to dinucleotide shuffled sequences using saliency maps for computing importance scores.

SFig. 2D Q-values – corrected with the Benjamini-Hochberg FDR procedure – of **SFig. 2C.**

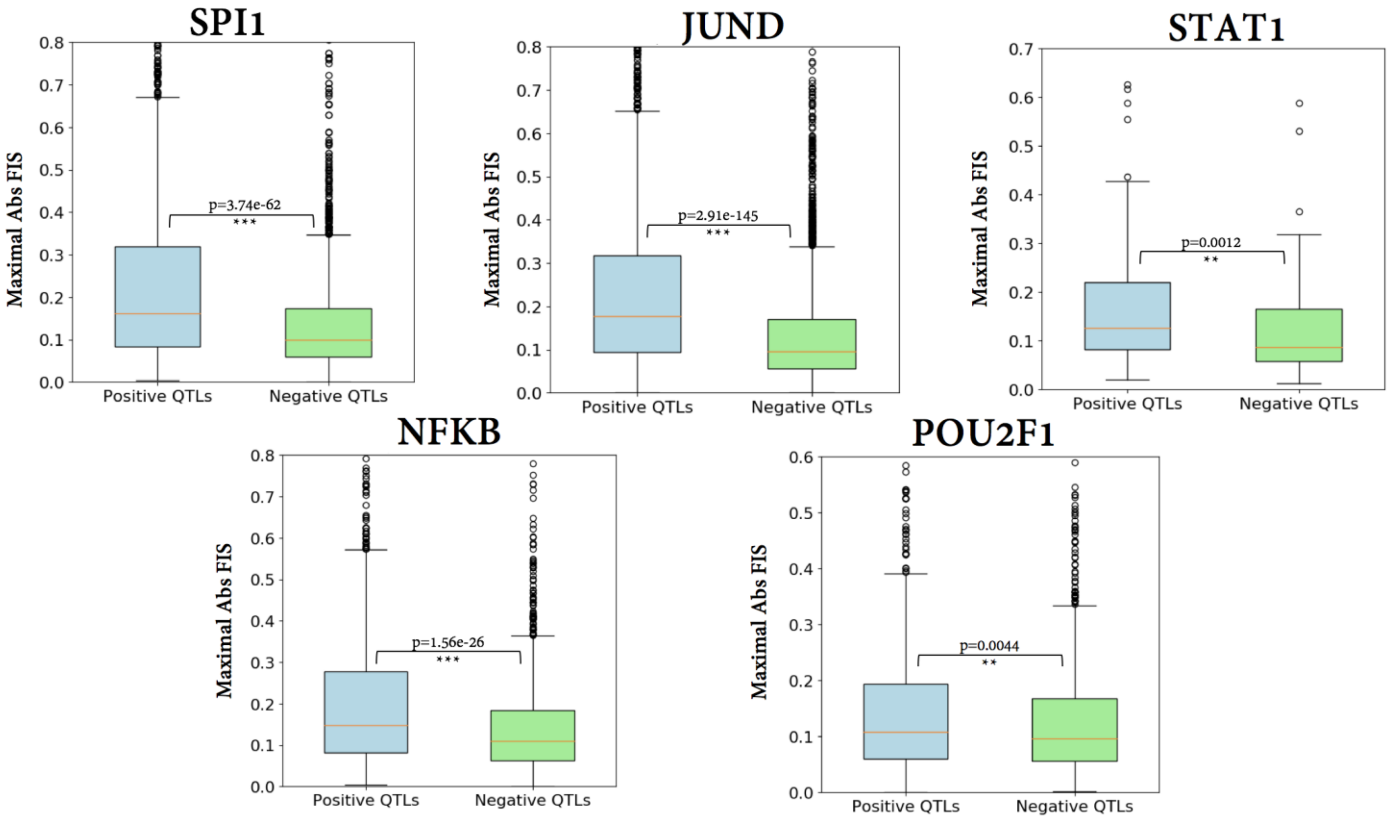

SFig. 3 Significant differences between positive (significant p<5e-5) and insignificant (p=1) bQTLs are recapitulated using importance scores computed with saliency maps, showing robustness of the method across multiple importance score methods.

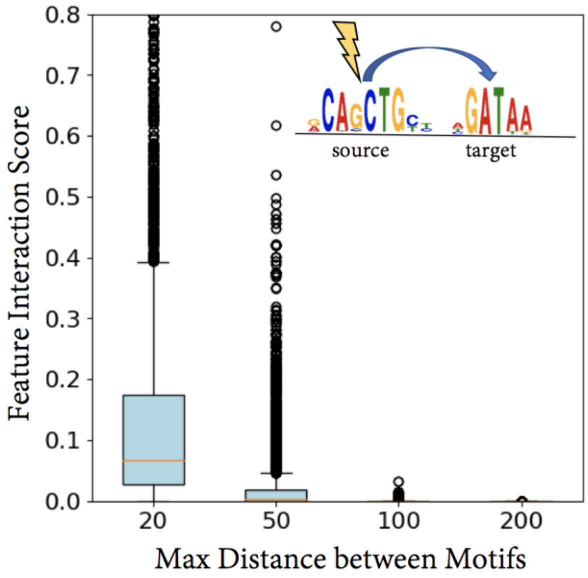

SFig. 4 Mutating TAL1 also has an effect on GATA1 motifs within 20bp in comparison to those that are greater than 20bp away, showing generally symmetric results to those in **Fig. 3B.**

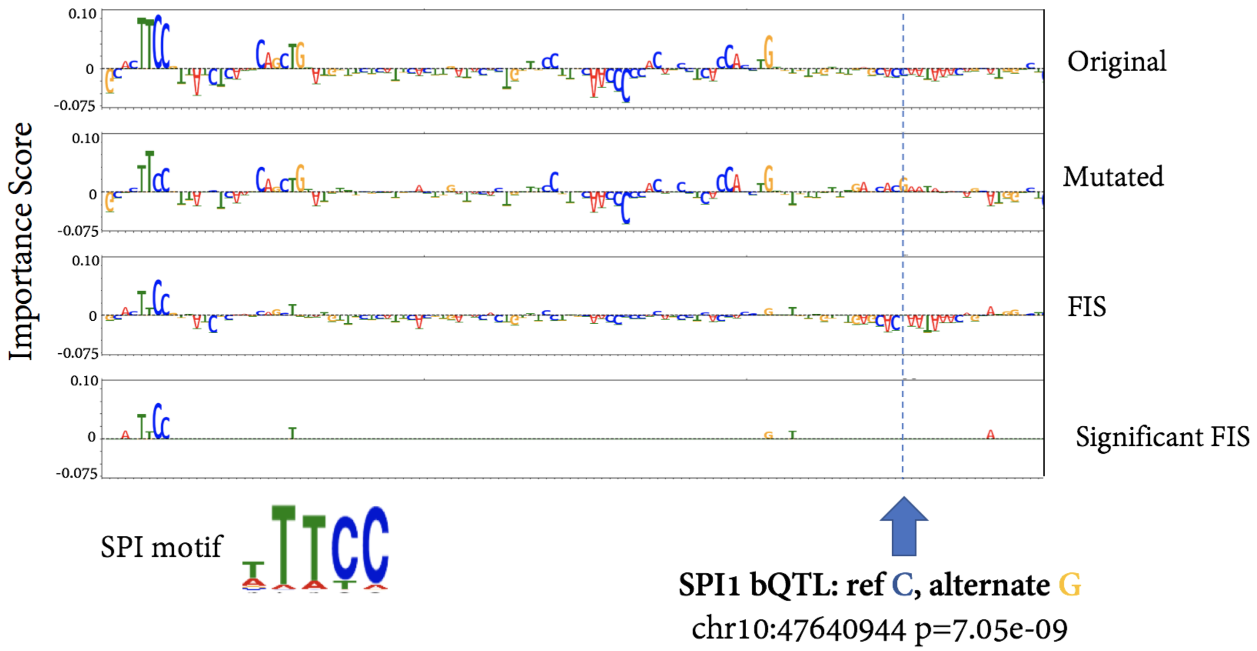

SFig. 5 This SPI1 QTL appears to modulate the strength of an SPI1 binding site 100 base pairs away from the actual variant site.

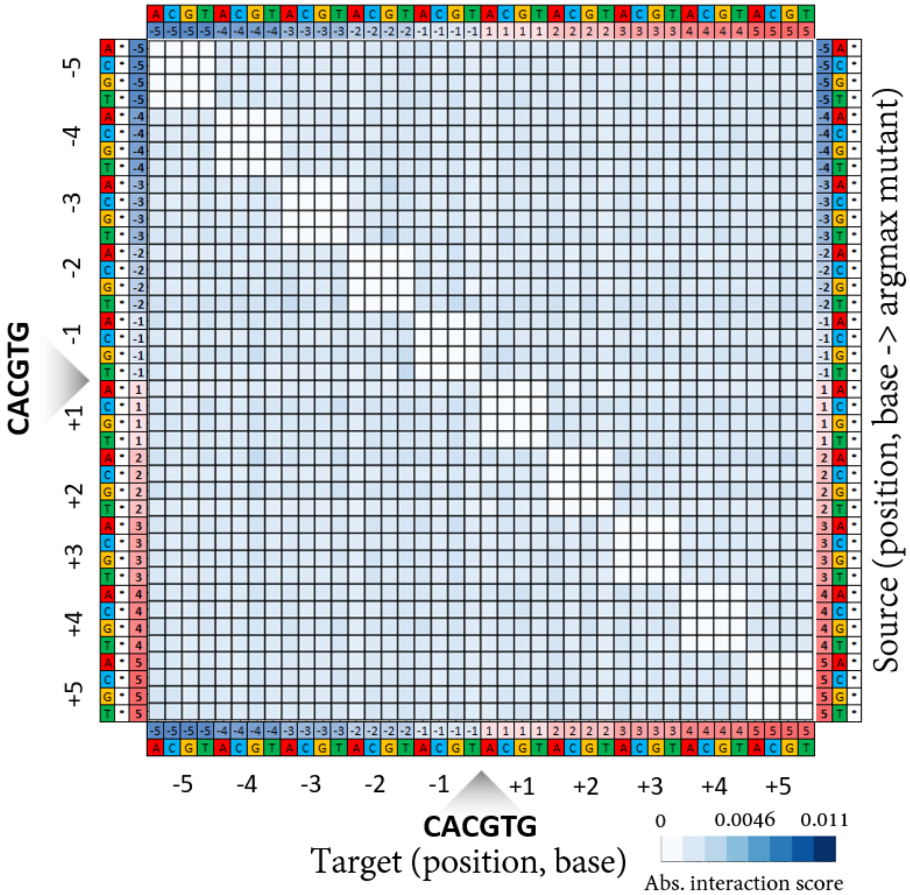

SFig. 6 We observe weak pairwise interactions between positions in the marginalized aggregate DFIM for Cbf1 across the 5K lowest binding affinity sequences. The rows correspond to (source position, source base, argmax mutant base). The columns correspond to (target position, target base).

References

1. Kheradpour, P. & Kellis, M. Systematic discovery and characterization of regulatory motifs in ENCODE TF binding experiments. *Nucleic Acids Res* **42,** 2976–2987 (2014).
2. Shrikumar, A., Greenside, P. & Kundaje, A. Learning Important Features Through Propagating Activation Differences. in **70,** 3145–3153 (Proceedings of Machine Learning Research, 2017).
3. Gerstein, M. B. *et al.* Architecture of the human regulatory network derived from ENCODE data. *Nature* **489,** 91–100 (2012).
4. Kellis, M. *et al.* Defining functional DNA elements in the human genome. *Proc Natl Acad Sci U S A* **111,** 6131–6138 (2014).
5. ENCODE Project Consortium. An integrated encyclopedia of DNA elements in the human genome. *Nature* **489,** 57–74 (2012).
6. Feng, J., Liu, T., Qin, B., Zhang, Y. & Liu, X. S. Identifying ChIP-seq enrichment using MACS. *Nat Protoc* **7,** 1728–1740 (2012).
7. Corces, M. R. *et al.* Lineage-specific and single-cell chromatin accessibility charts human hematopoiesis and leukemia evolution. *Nat Genet* **48,** 1193–1203 (2016).
8. Roadmap Epigenomics Consortium *et al.* Integrative analysis of 111 reference human epigenomes. *Nature* **518,** 317–330 (2015).
9. Tehranchi, A. K. *et al.* Pooled ChIP-Seq Links Variation in Transcription Factor Binding to Complex Disease Risk. *Cell* **165,** 730–741 (2016).
